# The gut microbiome composition and degradation enzymes activity of black Amur bream (*Megalobrama terminalis*) in response to breeding migratory behavior

**DOI:** 10.1002/ece3.7407

**Published:** 2021-04-04

**Authors:** Yaqiu Liu, Xinhui Li, Jie Li, Weitao Chen

**Affiliations:** ^1^ Pearl River Fisheries Research Institute Chinese Academy of Fishery Sciences Guangzhou China; ^2^ Scientific Observing and Experimental Station of Fishery Resources and Environment in Middle and Lower Reaches of Pearl River Ministry of Agriculture and Rural Affairs Guangzhou China

**Keywords:** enzymes, gut microbial community, *Megalobrama terminalis*, metabolism, migration

## Abstract

Black Amur bream (*Megalobrama terminalis*), a dominant species, resides in the Pearl River basin, known for its high plasticity in digestive ability. During spawning season, *M. terminalis* individuals with large body size and high fertility undergo a spawn migratory phase, while other smaller individuals prefer to settlement over migration. It is well known that gut microbial community often underpins the metabolic capability and regulates a wide variety of important functions in fish. However, little was known about how the gut microbiomes affect fish breeding migration. To investigate the variations in the gut microbiome of *M. terminalis* during the migration, we used high‐throughput 16S rRNA gene sequencing to reveal the distinct composition and diversity of the whole gut microbiome of migrated and nonmigrated population during period of peak reproduction, respectively. Our results indicated that nonmigrated population in estuary had a higher alpha diversity than that of migrated population in main stem. Additionally, an obvious abundant taxa shift between the gut microbiota community of nonmigrated and migrated *M. terminalis* was also observed. Change of dominant gut taxa from nonmigrated to migrated population was thought to be closely related to their degradation enzymes. Our results suggested that amino acid metabolism and lipid metabolism in migrated population were higher than that in nonmigrated population, providing a line of evidence for that *M. terminalis* change from partial herbivorous to partial carnivorous diet during breeding migration. We further concluded that, in order to digest foods of higher nutrition to supply energy to spawning migration, *M. terminalis* regulate activities of the gut microbiome and degradation enzymes, considered to be a key physiological strategy for reproduction.

## Graphical abstract

Current study is the first demonstration of gut microbiome in *Megalobrama terminalis* during the breeding migration. Significant differences of gut microbiota communities of *M. terminalis* between non‐migration and migration group have been found in our research. Gut microbiota of *M. terminalis* was found not only to aid in digestion, but also to play an important role in its metabolic capacity and endocrine system during the breeding migration.

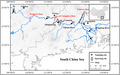

## 1 INTRODUCTION

The gut microbiota has been regarded as a key factor in the health and nutrition of their host (Wang et al., 2018). The composition of gut microbial communities can reinforce the metabolic capacity and provide a series of beneficial effects for their hosts, such as maintaining nutrient digestion, immune function, and resistance to pathogen invasion, and regulating energy absorption (Aidy et al., 2013; Viaud et al., 2013). Almost half of all vertebrate species were fish, which encompass a wide spectrum of host habitats, physiologies, and ecological strategies (Parris et al., 2016). Fish gut microbiota can contribute to digestion and affect the growth, reproduction, overall population dynamics, and vulnerability to disease of host fish (Ghanbari et al., 2015; Parris et al., 2016). A few gut microbiota can even metabolize a remarkable variety of substrates, such as microbial species isolated from the intestinal tracts of herbivorous fish species identified as the cellulolytic enzyme‐producing bacterial community (Li et al., 2016). In addition, food composition, physiological state, and environmental and genetic factors can affect gut microbial communities, thus affecting nutritional metabolism, immune regulation, and neuroendocrine system of the host (Meng et al., 2019). Fishelson et al. (1985) demonstrated that the distinct and diverse gut microbiota composition in herbivorous surgeon fish was closely associated with the trophic level of the host. Thus, either the diet category or the trophic level is the major factor that drives the composition and metabolism of the fish gut microbiota (Liu et al., 2016).

It is well recognized that the structure and composition of gut microbiota and their ecological function were strongly influenced by a range of factors, including the host genetics and physiology, living environment, and diet (Fishelson et al., 1985; Ley et al., 2008; Scott et al., 2013). The fish gut microbiota is closely related to the growth and development of fish (Ghanbari et al., 2015). To introduce how the conditions of the host fish and other factors affect the fish gut microbiota in return, Lin et al. (2014) found that the intestinal microbial community could be used as a method to reveal animal behavior. Currently, mice, macaques, chickens, earthworms, and termites have been used to successfully correlate the effects of intestinal microbial communities to host physiology (Drake et al., 2006; McKenna et al., 2008; Ohkuma & Brune, 2010; Torok et al., 2008; Zhang et al., 2009). Despite of the recognized important role of microbiomes in host ecology (Wong and Rawls, 2012), previous studies of fish gut microbiomes have focused primarily on commercial or model species host (Roeselers et al., 2011; Clements et al., 2014). On the contrary, the connection between gut microbial composition and fish physiology has been reported in only a few fish species and needs for further research to elucidate completely.

**FIGURE 1:**
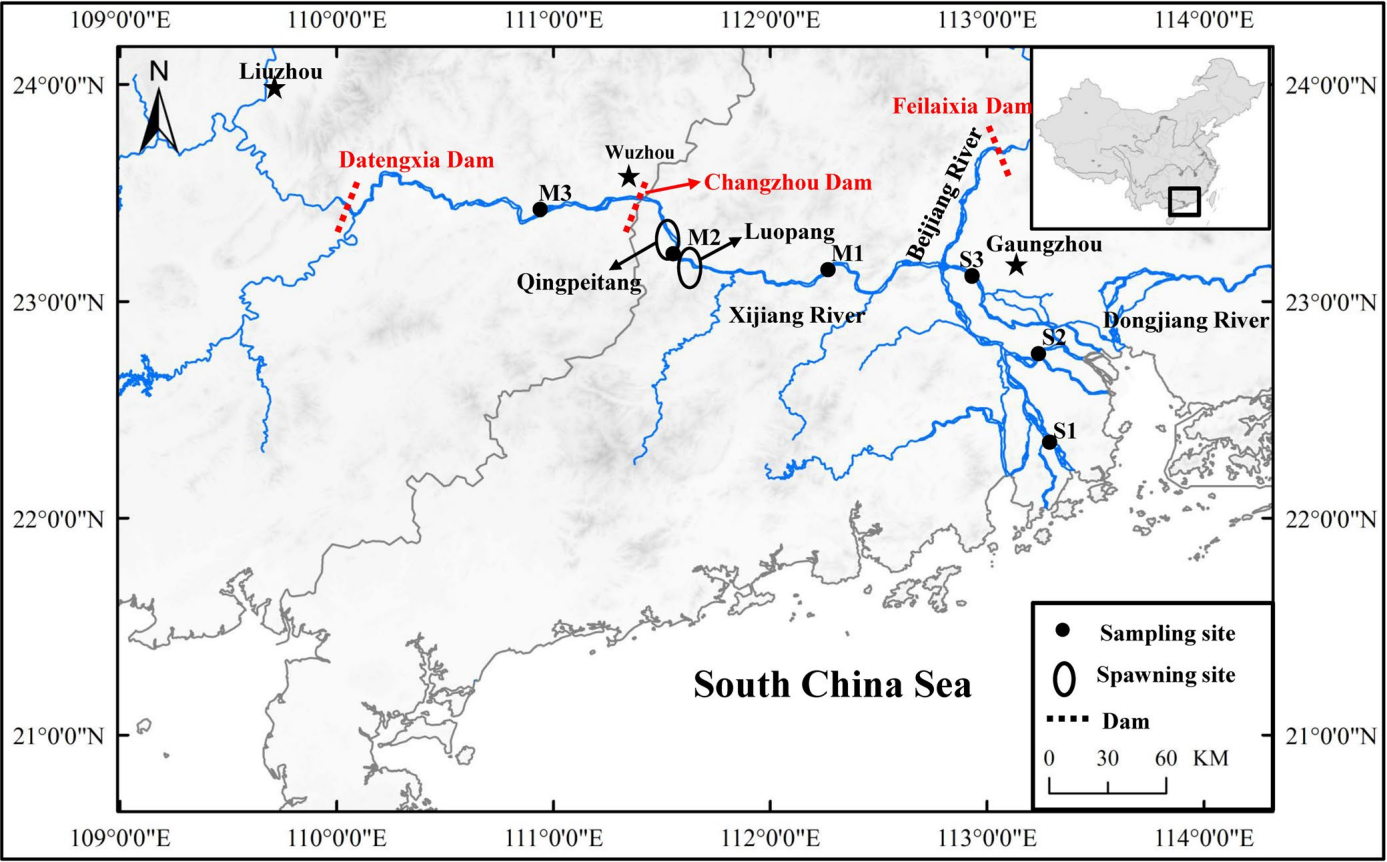
Sampling map showing the locations of *Megalobrama terminalis*. Black circles were shown as spawning ground of *M. terminalis* in Pearl River

The black Amur bream (*Megalobrama terminalis*) is a migratory fish species that lives in the middle and bottom of rivers, widely distributed in southern China drainages (Chen et al., 2020). It has been reported that the black Amur bream is one of the most important commercial fish species in the middle and lower reaches of the Pearl River, and the biomass of this species accounted for nearly 44.1% in all fish catches (Liu et al., 2020). Previous studies have shown that the *M. terminalis* growing and fattening in the estuary migrates nearly 250 km upstream from the estuary to the spawning grounds (Luopang and Qingpeitang) (Figure 1), where the water is lotic and the riverbed has an abundance of rocks. Liu et al. (2021) found that the abundance of spawning *M. terminalis* occurs from late June to mid‐July. During migration, gonads of *M. terminalis* revived. In our previous study, we found that not all the *M. terminalis* individuals in estuary participated in breeding migration (Liu et al., 2021). Usually, for sexually mature individuals with large body size and high fertility, the cost of migration is less than of settlement; thus, the migration was deemed to be obviously "profitable" (Wysujack et al., 2010). Migration has evolved repeatedly in animals, and many migratory fish reproduction strategies are found across the tree of life that increased migration efficiency (Burns & Bloom, 2020). Caudill et al. (2007) suggested that fish can adjust its own physiological behavior to meet the energy needs of breeding migration. Barneche et al. (2018) also found that fish body size determines total migration reproductive‐energy output. Change of body length limit, swimming speed, osmotic pressure regulation, hormone, diet, sexual maturity, and gonad development which were regarded as a preparation process before fish migration (Ojima & Iwata, 2007; Saborido‐Rey et al., 2004; Xia et al., 2017). Larger individuals reproduce disproportionately more than smaller individuals in not only fecundity but also total reproductive energy (Barneche et al., 2018; Burns & Bloom, 2020).

A recent study showed that there had been a shift in the diet of black Amur bream during gonad development period, which exhibits the different food preferences of both juveniles and adults (Xia et al., 2017). Furthermore, some studies have indicated that *M. terminalis* is an omnivorous fish with high plasticity in digestive ability (Liu et al., 2020; Xia et al., 2017). To date, most studies on black Amur bream have focused on larval resources, feeding habits, ecological investigation of spawning grounds, and gonad development (Li et al., 2014; Liu et al., 2019; Tan et al., 2009; Wang et al., 2010; Xia et al., 2017). However, the gut flora diversity and composition of the black Amur bream are poorly understood. Given the general close relation between the gut microbiome and the host, we hypothesized: (a) *M. terminalis* can change its gut microbiome communities during reproductive migration period; (b) some differences between microbiome composition of migrated and nonmigrated *M. terminalis* individuals exist. To demonstrate our hypothesis, we investigated that diversity of gut microorganisms of wild *M. terminalis* stock in fattening ground, migration routes, and spawning ground during period of peak reproduction to explain how they effectively ensure their digestion of food and disease resistance during reproductive migration. Additionally, due to the closure of the Changzhou Dam, wild *M. terminalis* stock has shown a decreasing trend (Chen et al., 2020; Liu et al., 2021), gut floral communities of *M. terminalis* have been identified, which also provide theoretical support for its conservation biology.

## 2 MATERIALS AND METHODS

### 2.1 Fishing sampling

In this study, a total of 180 M*. terminalis* specimens were collected from six localities using circular cast nets (16 m diameter, mesh size 3 cm) in the main stem and estuary of the Pearl River during June and July 2018 (Figure 1). Water temperatures, salinity, dissolved oxygen (DO), and pH of sampling sites were measured with (HQ30, Hach Company, Loveland, CO, USA). The sampling variables including date of collection, location, water temperatures, salinity, pH, and DO are provided in Table 1. Standard length (SL, to the nearest 1 mm): the length from the tip of the mandible to the base of the caudal fin; body weight (W_t_, to the nearest 1 g), eviscerated weight (EW, to the nearest 1 g), gonad and liver weights (GW and LW, respectively, to the nearest 0.01 g) were measured. The gonadal development stages of *M. terminalis* were categorized into immaturity and maturity based on gonad morphological characteristics, as described by Liu et al. (2019). The sex mature ratio (SMR = 100 × number of mature/total investigated quantity) and the gonadosomatic index (GSI = 100 × GW/EW) were estimated as an indicator of fish reproductive periods. The hepatosomatic index (HSI = 100 × LW/EW) and fatness (K = 100 × W_t_/SL^3^) were regarded as bioenergetic indices to evaluate fish conditions. To investigate the fish gut flora, five fish were randomly selected for sequencing from six sample sites in two populations: nonmigrated (S1, S2, S3) and migrated population (M1, M2, M3). Selected fish were stunned and decapitated quickly. The body surface was wiped to remove redundant mucus. All instruments, surfaces of each fish, were treated with 70% ethanol to ensure the skin surface, and instruments were sterilized before dissection. After opening the body cavity, the entire intestinal tract and its contents were aseptically removed from each individual fish. Approximately 0.4 g of gut contents was extracted for DNA extraction, and 0.2 g of gut contents for enzymatic analysis; all the contents samples were then stored at −40°C until test.

**TABLE 1:** Basic environmental information, biological information, and alpha diversity results of gut microbial community pertaining for the different group studied of *Megalobrama terminalis*

		Nonmigration				Migration			
		S1	S2	S3	Overall	M1	M2	M3	Overall
Environmental information	Sample period	July 23–29	July 17–23	July 11–16		July 4–10	June 27–July 3	June 20 to 26	
	Temperature (℃)	28.8 ± 0.2	28.1 ± 0.3	28.2 ± 0.4	28.4 ± 0.3	28.7 ± 0.4	28.6 ± 0.3	28.9 ± 0.3	28.7 ± 0.3
	Salinity (‰)	0.15 ± 0.03^b^	0.09 ± 0.02^ab^	0.08 ± 0.02^ab^	0.11 ± 0.02*	0.01 ± 0.00^a^	0.01 ± 0.00^a^	0.01 ± 0.00^a^	0.01 ± 0.00
	pH	8.1 ± 0.1	8.1 ± 0.1	7.9 ± 0.2	8.0 ± 0.1	8.2 ± 0.2	8.3 ± 0.2	7.8 ± 0.2	8.1 ± 0.2
	DO (mg/L)	6.6 ± 0.1^a^	6.3 ± 0.2^a^	6.8 ± 0.2^ab^	6.6 ± 0.2	7.1 ± 0.1^b^	7.7 ± 0.2^b^	6.9 ± 0.2^ab^	7.2 ± 0.2*
Biological information	SL ± *SD*	174 ± 22.6^a^	186 ± 16.6^a^	225 ± 22.3^ab^	205 ± 18.4	244 ± 28.4^b^	265 ± 21.1^b^	256 ± 14.4^b^	255 ± 21.8*
	W_t_ ± *SD*	96.8 ± 21.3^a^	120 ± 33.2^a^	257 ± 29.1^b^	158 ± 27.5	359 ± 25.5^c^	395 ± 35.1^c^	352 ± 26.3^c^	369 ± 28.4*
	GSI (%)	0.7 ± 0.1^a^	0.9 ± 0.1^a^	5.3 ± 1.3^b^	3.4 ± 0.5	5.9 ± 1.4^b^	8.4 ± 2.3^b^	5.6 ± 0.9^b^	6.6 ± 1.5*
	HSI (%)	1.4 ± 0.12^a^	2.2 ± 0.15^b^	2.6 ± 0.13^c^	2.1 ± 0.14*	2.1 ± 0.12^bc^	1.2 ± 0.11^a^	1.0 ± 0.11^a^	1.4 ± 0.11
	K	1.7 ± 0.12^a^	2.0 ± 0.16^ab^	2.1 ± 0.21^ab^	1.9 ± 0.16	2.2 ± 0.24^b^	2.3 ± 0.23^b^	2.2 ± 0.16^b^	2.2 ± 0.21*
	Sex mature ratio (%)	0	0	23.7	7.9	83.9	100	100	94.6
Alpha diversity estimates	OTUs	1,261 ± 348.83^b^	1,876 ± 293.36^b^	1,305 ± 617.70^ab^	1,478 ± 405.61*	1,226 ± 392.15^ab^	802 ± 224.39^a^	576 ± 174.29^a^	868 ± 232.56
	Observed_species	1,258 ± 156.72^a^	1,663 ± 228.77^a^	1,304 ± 272.17^a^	1,408 ± 232.11*	1,184 ± 146.49^a^	732 ± 112.91^ab^	586 ± 74.97^b^	834 ± 117.21
	Chao1	1,396.89 ± 168.89^a^	1,881.23 ± 238.08^a^	1,512.20 ± 282.11^a^	1,596 ± 230.82*	1,383.45 ± 146.75^ab^	852.21 ± 124.78^b^	749.69 ± 117.29^b^	995.11 ± 128.97
	Shannon	7.12 ± 0.58^a^	6.77 ± 0.61^a^	5.27 ± 0.67^ab^	6.39 ± 0.63*	4.82 ± 0.24^b^	4.87 ± 0.37^b^	4.38 ± 0.27^b^	4.69 ± 0.29

Different superscript letters indicate significant differences in different groups, *p* < .05.

Abbreviations: DO, dissolved oxygen; GSI, gonadosomatic index; HSI, hepatosomatic index; K, Fatness; SL, standard length; W_t_, body weight.

* Means significant difference between two populations, *p* < .05.

### 2.2 DNA extraction, amplification, and sequencing

Approximately 0.2 g of each sample was extracted using a QIAamp DNA Stool Mini Kit (Qiagen, Valencia, USA) following the manufacturer's recommendations. All DNA extracts were stored at −40°C until required. The quality and integrity of each DNA concentration and purity were monitored on 1% agarose gels. Total DNA from the gut of different groups of black Amur bream was sent to Novogene Bioinformatics Technology, Co., Ltd., Beijing, China for further sequencing analysis.

The 16S rRNA genes of distinct regions were amplified using the specific primer V4 hypervariable region (515F–806R) with the barcode. All PCR reactions were conducted in 30 μl reactions with 15 μl of High‐Fidelity PCR Master Mix (New England Biolabs), 0.2 μM of forward and reverse primers, and approximately 10 ng template DNA. Thermal cycling consisted of initial denaturation at 98°C for 1 min, followed by 30 cycles of denaturation at 98°C for 10 s, annealing at 50°C for 30 s, elongation at 72°C for 30 s, followed by 5 min at 72°C. The same volume of 1 × loading buffer (contained SYB green) with PCR products was used for electrophoresis on 2% agarose gel for detection. DNA concentration was measured using a NanoDrop ND‐2000 spectrophotometer (Thermo Scientific, USA). Sequencing libraries were generated using the Ion Plus Fragment Library Kit 48 rxns (Thermo Scientific, USA) following the manufacturer's instructions. The library quality was assessed on a Qubit@ 2.0 Fluorometer (Thermo Scientific, USA). Finally, the library was sequenced on an Ion S5 TM XL platform, and 400 bp/600 bp single‐end reads were generated.

### 2.3 Enzyme assays

The samples were removed from the freezer and placed on ice to thaw. After thawing was complete, the samples were homogenized with an F6/10 Fluko homogenizer at 12,000 *g* for 2 min on ice in 0.2 M NaCl (Gawlicka *et al*., 2000). The resulting homogenate was centrifuged with a cryogenic ultracentrifuge, and the supernatant was used to determine the digestive enzyme activities and soluble proteins.

The activities of trypsin were measured with trypsin assay kit (NO. A080‐2, Nanjing Jiancheng Bioengineering Institute, P.R. China). In the assay kit, trypsin catalyzes the hydrolysis of the substrate arginine ethyl ester chain, absorbance increase was detected at *λ* = 253 nm. Amylase was measured with Amylase assay kit (NO: C016, Nanjing Jiancheng Bioengineering Institute, P.R. China). In the assay kit, starch was hydrolyzed to produce glucose, maltose, and dextrin by amylase, probe absorbance of blue complex chromophore was measured at *λ* = 660 nm after add iodine. Cellulase was measured with cellulose assay kit (NO: A138, Nanjing Jiancheng Bioengineering Institute, P.R. China). In the assay kit, cellulase hydrolyzes cellulose to produce cellulosic disaccharides, and glucose and other reducing sugars can reduce 3, 5‐dinitrosalicylic acid (DNS) under alkaline conditions to produce red‐brown ammonoids, and absorbance increases were detected at *λ* = 550 nm. Lipase was measured with a lipase assay kit (NO: A054‐2, Nanjing Jiancheng Bioengineering Institute, P.R. China). In the assay, 1, 2‐laurelglycerol‐3‐glutaraldehyde‐6′‐methyl resorufin could be catalyzed by lipase, and chromophore variation was detected at *λ* = 580 nm.

The specific activity was expressed as milli‐units per milligram of protein or units per milligram of protein (mU/mg protein or U/mg protein). The soluble protein (mg/mL) contents were determined with the Bradford method using bovine serum albumin as the standard (0.563 g/L) (Bradford, 1976). All assays were performed on duplicate samples using an Infinite M200 Pro Tecan Sunrise (Tecan, Männedorf, Switzerland). UV‐permeable Corning 96‐well microplates (Corning Incorporated, Corning, USA) were used for all assays. All reactions were run at the saturating substrate concentrations determined for each enzyme.

### 2.4 Data analysis

Paired‐end reads were merged from the original DNA fragments using FLASH software and assigned to samples based on their unique barcode, and truncated by cutting off the barcode and primer sequence. Quality filtering of the raw reads was performed under specific filtering conditions to obtain high‐quality clean reads according to the Cutadapt quality‐controlled process (Martin, 2011). The tags were compared with the reference database using the UCHIME algorithm to detect chimera sequences (Edgar et al., 2011; Haas et al., 2011). Sequence analysis was performed using Uparse software (Edgar, 2013). Sequences with equal or greater than 97% similarity were assigned to the same operational taxonomic unit (OTU). Representative sequences for each OTU were screened for further annotation. The shared and unique OTUs of different groups were also represented by a Scale‐Venn diagram using Euler APE.

To compute the alpha diversity of fish gut microbiota, three metrics were calculated: chao1 estimates for species abundance; observed species estimates for the number of unique OTUs found in each sample, and the Shannon index. Rarefaction curves were generated based on these three metrics. QIIME calculates unweighted UniFrac, which is a phylogenetic measure of beta diversity. We used unweighted UniFrac for principal coordinate analysis (PCoA). PCoA helps to obtain principal coordinates and allows their visualization from complex, multidimensional data. It transforms a distance matrix to a new set of orthogonal axes. The maximum variation factor is demonstrated by the first principal coordinate, the second maximum by the second principal coordinate. Permutation multivariate analysis of variance (PERMANOVA) was used to test the statistical significance of six sample sites in two populations: nonmigrated (S1, S2, S3) and migrated population (M1, M2, M3) (Anderson, 2001; Stat et al., 2013). MetaStat method was used to compare species abundance between groups and select species with significant differences (Robert et al., 2009). To explore the metabolic activity of the bacterial communities on the gut contents of different groups, Phylogenetic Investigation of Communities by Reconstruction of Unobserved States (PICRUSt) was used to construct the Kyoto Encyclopedia of Genes and Genomes (KEGG) pathway (Langille et al., 2013). The functions were analyzed at levels of 2 and 3.

STATISTICA 6.0 (StatSoft, Inc., Tulsa, OK, USA) was used for statistical analysis of the recorded data. The normality of the data and homogeneity of variance were assessed with the Kolmogorov–Smirnov test and Leven's test, respectively. In the nonmigrated (S1, S2, S3) and migrated population (M1, M2, M3), enzymatic activities were determined by one‐way analysis of variance (ANOVA). All data are expressed as means ± *SD* (*n* = 5). To better understand the relationship between gut microbial diversity and its enzymes activity in fishes with different trophic levels, the canonical correspondence analysis (CCA) was conducted. Here, we used the *R* implementation of the procedure (version 1.1‐3).

## 3 RESULTS

### 3.1 Basic biological parameters of fish

Salinity showed a decreasing trend from nonmigrated to migrated population, salinity of S1 group was significant higher than that of M1, M2, and M3 group (*p* < .05) (Table 1). The basic data for fish samples from the six sample sites are shown in Table 1. SL, W_t_, GSI, K, and SMR showed an increasing trend from nonmigrated to migrated population. SL, W_t_, and GSI of the black Amur bream in S1 and S2 groups were much lower than M1, M2, and M3 groups (*p* < .05). Nevertheless, HSI was observed to first rise then descend from nonmigrated to migrated population. S3 group had a highest HSI among all the groups. Meanwhile, the rate of mature individuals increased gradually from the estuary to main stem (Table 1).

### 3.2 Microbial complexity of fish gut flora

A total of 55,942 quality‐filtered sequences were obtained from each sample. With a 97% consistency (Identity), the sequences were grouped into a total of 8,709 OTUs. For the six groups listed in Table 1, gut microbial complexity was evaluated based on alpha diversity (OTU number, species, Chao1, and Shannon index) and indicated significant variation (Table 1). S2 group had the maximum alpha diversity indices, followed by S1, S2, M1, and M2 groups. M3 group exhibited the lowest alpha diversity indices. Our results showed that nonmigrated population in estuary had a higher alpha diversity than that of migrated population in main stem.

### 3.3 Comparison of the bacterial community in fish gut

**FIGURE 2:**
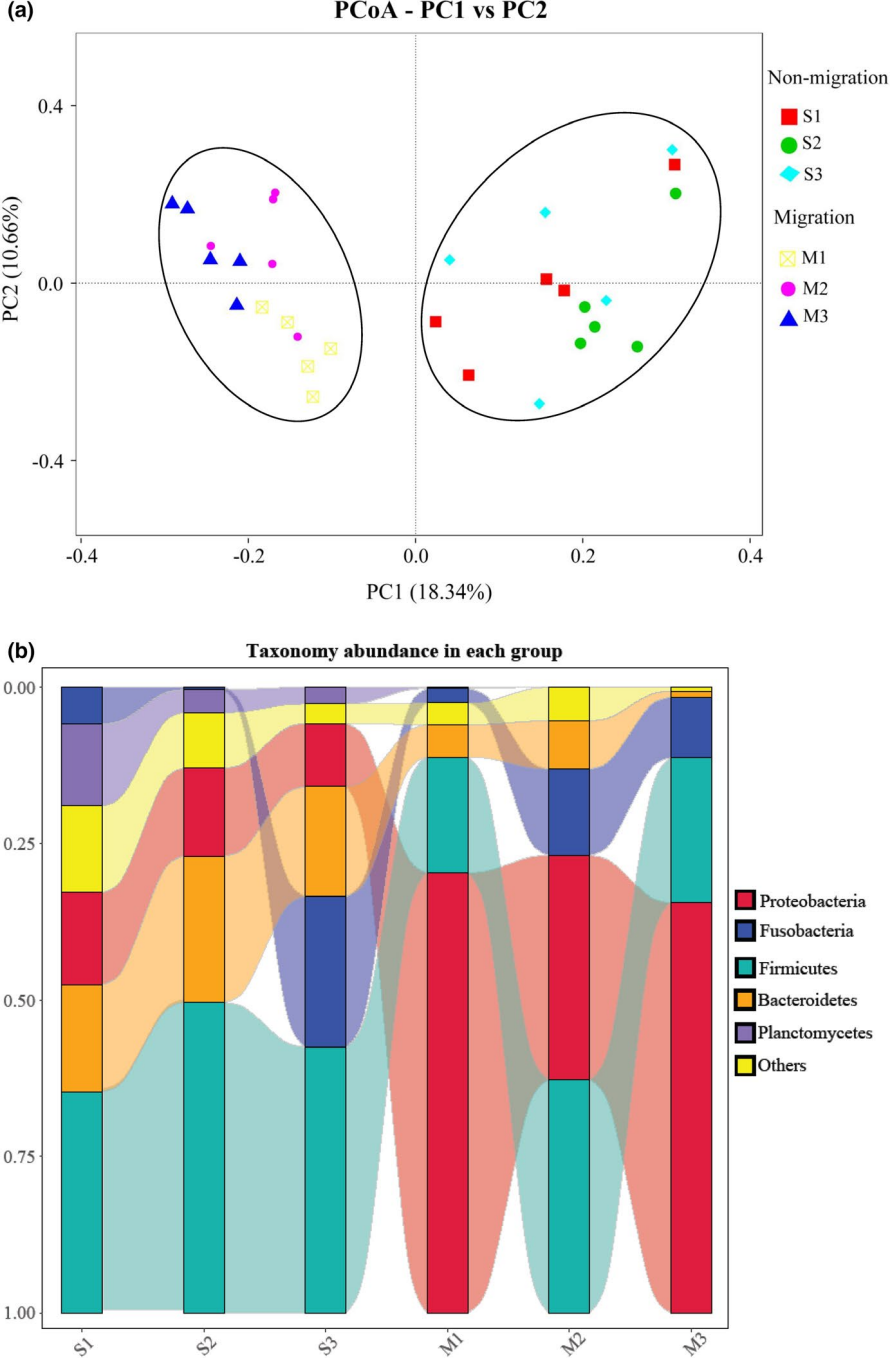
Comparison of the bacterial community in the different group studied of *Megalobrama terminalis*. (a) Principal coordinates analysis (PCoA) of bacterial community compositions the different group studied of *M. terminalis* based on the unweighted UniFrac distance matrix. The individual samples are color‐coordinated according to the different group. (b) Dominant gut microbiota composition in the different *M. terminalis* groups at phylum level. Each bar represents average relative abundance of each bacterial taxon within a group at phylum level

To compare the similarity of microbial community composition among different group, a PCoA was applied based on the unweighted UniFrac metrics. As shown in Figure 2a, a separation of the community composition between nonmigrated and migrated population was conspicuous. The community composition among S1, S2, S3 groups on PCoA scores showed more similar, that separated from community composition in the M1, M2, M3 group that formed a cluster. Samples in M2 group were located in middle of M1 and M3 groups. This clustering pattern was affected by spatial differences and gonadal maturation. However, PCoA1 and PCoA2 only explained for 18.34% and 10.66% of the total variance indicating that the fish gut bacterial community was influenced by complex factors rather than single factors. Approximately 99% of the total bacterial abundance was classified by 49 phyla and 861 genera. The most abundant taxa of bacteria in each investigated group of fish were observed at the phylum level (Figure 2b). At the phylum level, Firmicutes was the most abundant in the S1 (35.35%), S2 (49.62%), and S3 (42.51%) group, whereas the most abundant phylum of the M1 (70.35%) and M3 (65.59%) group was the Proteobacteria. The abundance of Firmicutes (35.91%) and Proteobacteria (37.23%) in M2 group was similar. Dominant microbiota (abundance > 15%) of *M. terminalis* in the nonmigrated population (S1, S2, S3) and migrated population (M1, M2, M3) showed significant differences in Firmicutes, Bacteroidetes, and Proteobacteria (*p* < .05). Results from the PERMANOVA analysis shown in Table 2 revealed significant differences between different groups. The difference between nonmigrated and migrated population was significant (*p* < .01). However, the correlation between each pair of S1, S2, and S3 group in nonmigrated population is not significant (*p* > .05). The similar result was also observed in M1, M2 and M3 group in migrated population. In general, an obvious abundant taxa shift between the gut microbiota community of nonmigrated and migrated *M. terminalis* was also observed.

**TABLE 2:** PERMANOVA analysis of the unweighted UniFrac for the different group studied of *Megalobrama terminalis*

Pair‐wise test	*p*
Nonmigration versus Migration	.001**
S1–S2	.329
S1–S3	.746
S1‐M1	.009**
S1‐M2	.007**
S1‐M3	.008**
S2–S3	.289
S2‐M1	.009**
S2‐M2	.011*
S2‐M3	.012*
S3‐M1	.008**
S3‐M2	.011*
S3‐M3	.008**
M1‐M2	.192
M1‐M3	.478
M2‐M3	.295

* Means significant difference between two populations (*p* < .05).

** Means very significant difference between two populations (*p* < .01).

### 3.4 Shared and unique microbial populations

**FIGURE 3:**
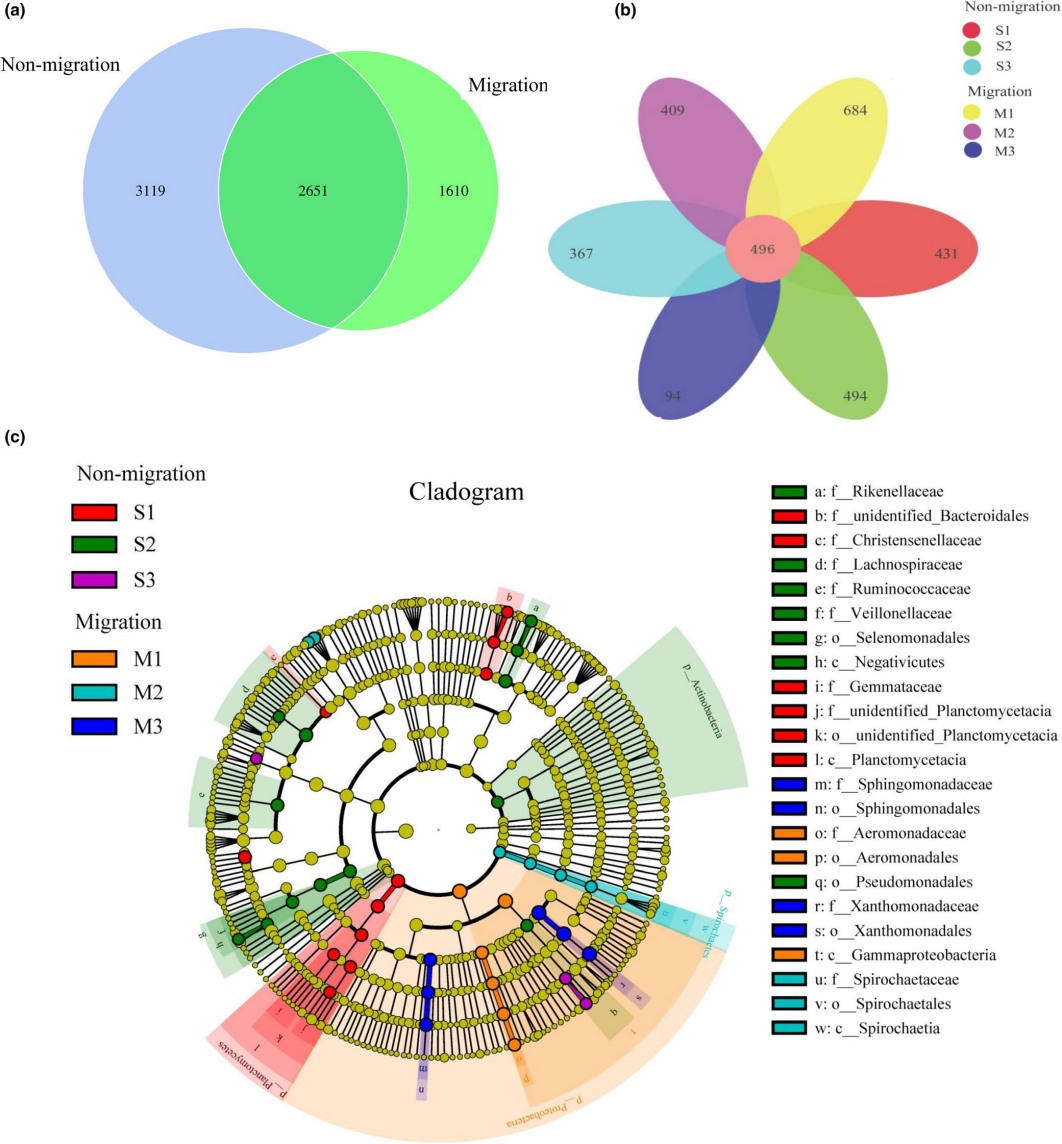
Shared and unique gut microbial populations in the different group studied of *Megalobrama terminalis*. (a) Venn diagram displays the number of shared and unique OTUs between *M. terminalis* nonmigration and migration population. (b) Venn diagram displays the number of shared and unique OTUs among six groups distributed in migration channel of *M. terminalis*. (c) Cladogram indicating LEfSe results presenting the recognized OTUs distributed according to phylogenetic characteristics around the circle. The dots in the center showed the OTUs at phylum level, whereas the outer circle of dots showed the OTUs at species level. The color of the dots and sectors present most abundant OTUs different groups studied of *M. terminalis* respectively. Yellow color indicates OTUs that showed similar abundance in all compartments. The colored sectors give information on phylum (full name in outermost circle, given only for phylum showing significant difference between groups, class, order, family that were significantly different between groups are showed at the right side of figure)

**FIGURE 4:**
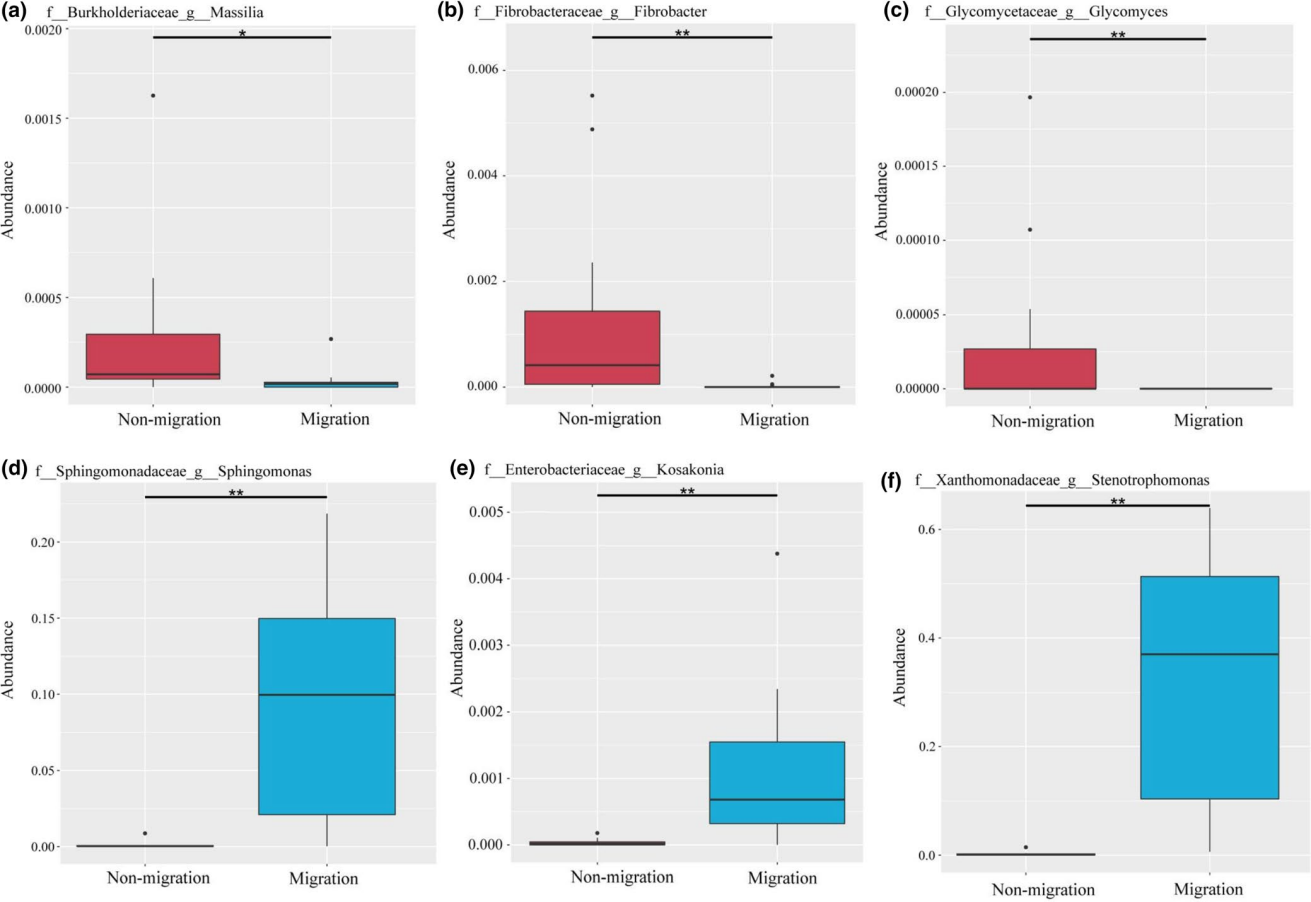
MetaStat analysis of the relative abundance of gut microbiota between nonmigrated and migrated *Megalobrama terminalis* population in genera level. (a) The relative abundance of *Massilia* between nonmigrated and migrated *M. terminalis* population. (b) The relative abundance of *Fibrobacter* between nonmigrated and migrated *M. terminalis* population. (c) The relative abundance of *Glycomyces* between nonmigrated and migrated *M. terminalis* population. (d) The relative abundance of *Sphingonmonas* between nonmigrated and migrated *M. terminalis* population. (e) The relative abundance of *Kosakonia* between nonmigrated and migrated *M. terminalis* population. (f) The relative abundance of *Stenotrophomonas* between nonmigrated and migrated *M. terminalis* population. "*" means significant difference between two populations (*p* < .05), "**" means very significant difference between two populations (*p* < .01)

To investigate the microbial community of gut samples from different groups, the shared and unique OTUs were constructed and visualized using a Venn diagram. A total of 5,790 and 4,261 OTUs were observed on nonmigration and migration population, respectively (Figure 3a). The nonmigration and migration population shared 2,651 OTUs, whereas the migration population shared fewer unique OTUs than that in the nonmigration population. The number of common OTUs presented in all groups was 496, and unique OTUs for each group varied from 94 to 684 (Figure 3b). M3 exhibited the lowest number of unique OTUs, whereas M1 had the largest number of unique OTUs. Linear discriminant analysis effect size (LEfSe) was used to characterize the microbial communities, and significant differences in abundances of different groups were observed (Figure 3c). The result showed that the class Planctomycetacia was significantly different in S1 compared that in the other groups. The abundance of order Selenomonadale and Pseudomonadales was observed significant higher in S2 group than that in other groups. Compared to the groups, M1 showed significantly higher abundance of family Aeromonadaceae, whereas order Spirochaetales was in M2 group significantly different from other groups. Compared to the other groups, the M3 group showed significantly higher abundance of the family Xanthomonadaceae and Sphingomonadaceae. MetaStat was used to analyze the relative abundance between nonmigrated and migrated populations in genera level. The result indicated that abundance of genera: *Massilia*, *Fibrobacter,* and *Glycomyces* in nonmigrated population were significantly higher than that in migrated populations (*p* < .05), while genera: *Kosakonia*, *Sphingomonas,* and *Stenotrophomonas* showed the opposite results (Figure 4).

### 3.5 Predicted gut microflora function using PICRUSt

**FIGURE 5:**
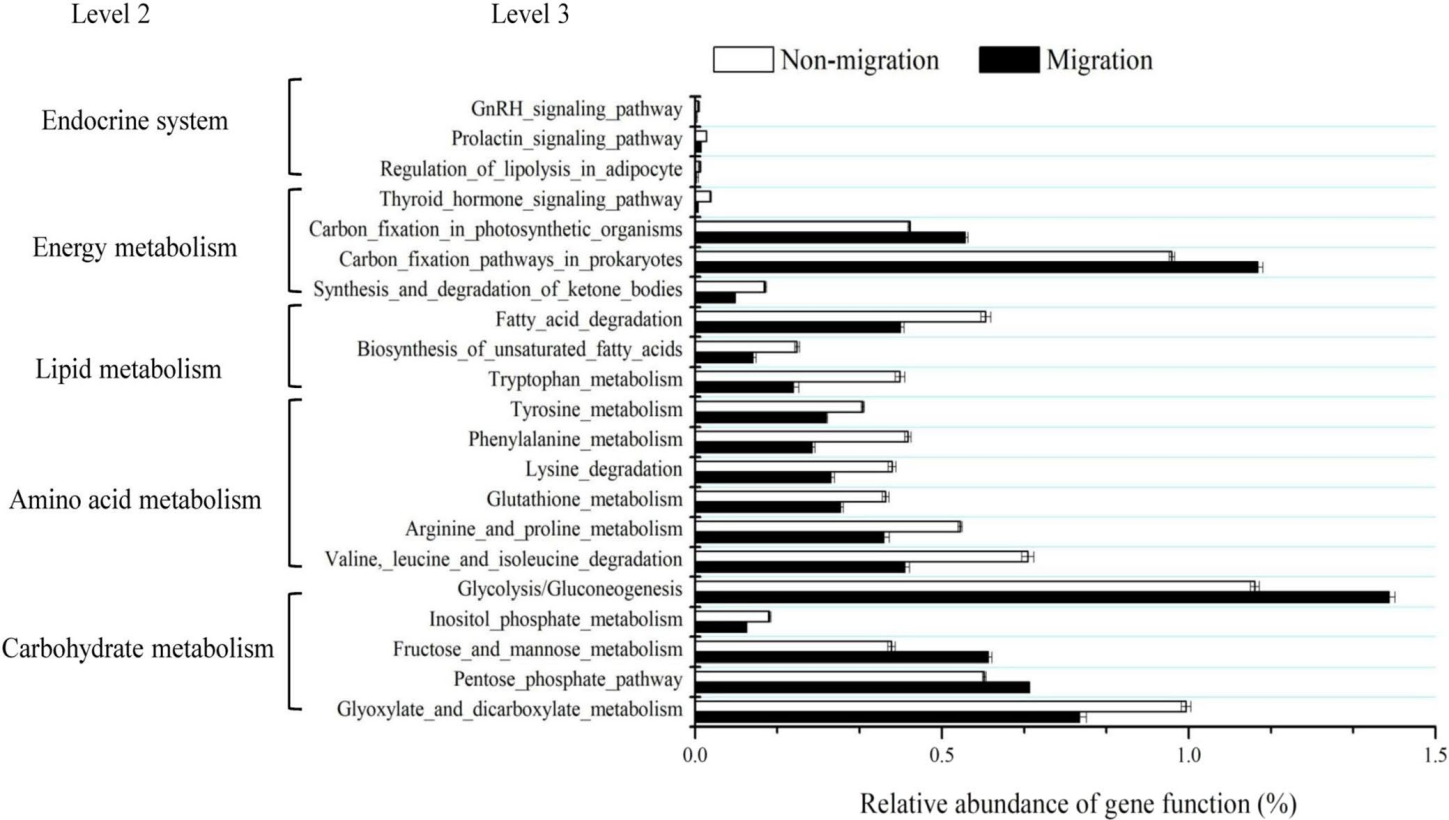
Comparison of the relative abundance of PICRUSt functional analysis of between nonmigrated and migrated *Megalobrama terminalis* population. Significant differences between nonmigrated and migrated *M. terminalis* population in gene categories at level 3 (*t* test, *p* < .05)

PICRUSt analyses indicated that *M. terminalis* in different population exhibited similar gene functions at levels 2 and 3 (Figure 5). The relative abundance of 21 genes for carbohydrate‐related metabolism, amino acid metabolism, lipid metabolism, energy metabolism, and the endocrine system showed statistically significant differences (*p* < .05 by *t* test) between migrated and nonmigrated population, as shown in Figure 5. The relative abundance for amino acid metabolism, lipid metabolism, and endocrine system in migrated population was higher than that in nonmigrated population. The gut microbiome predicted functions helped to elucidate the physiological features and metabolism capability during the fish breeding migration.

### 3.6 degradation enzymes

**FIGURE 6:**
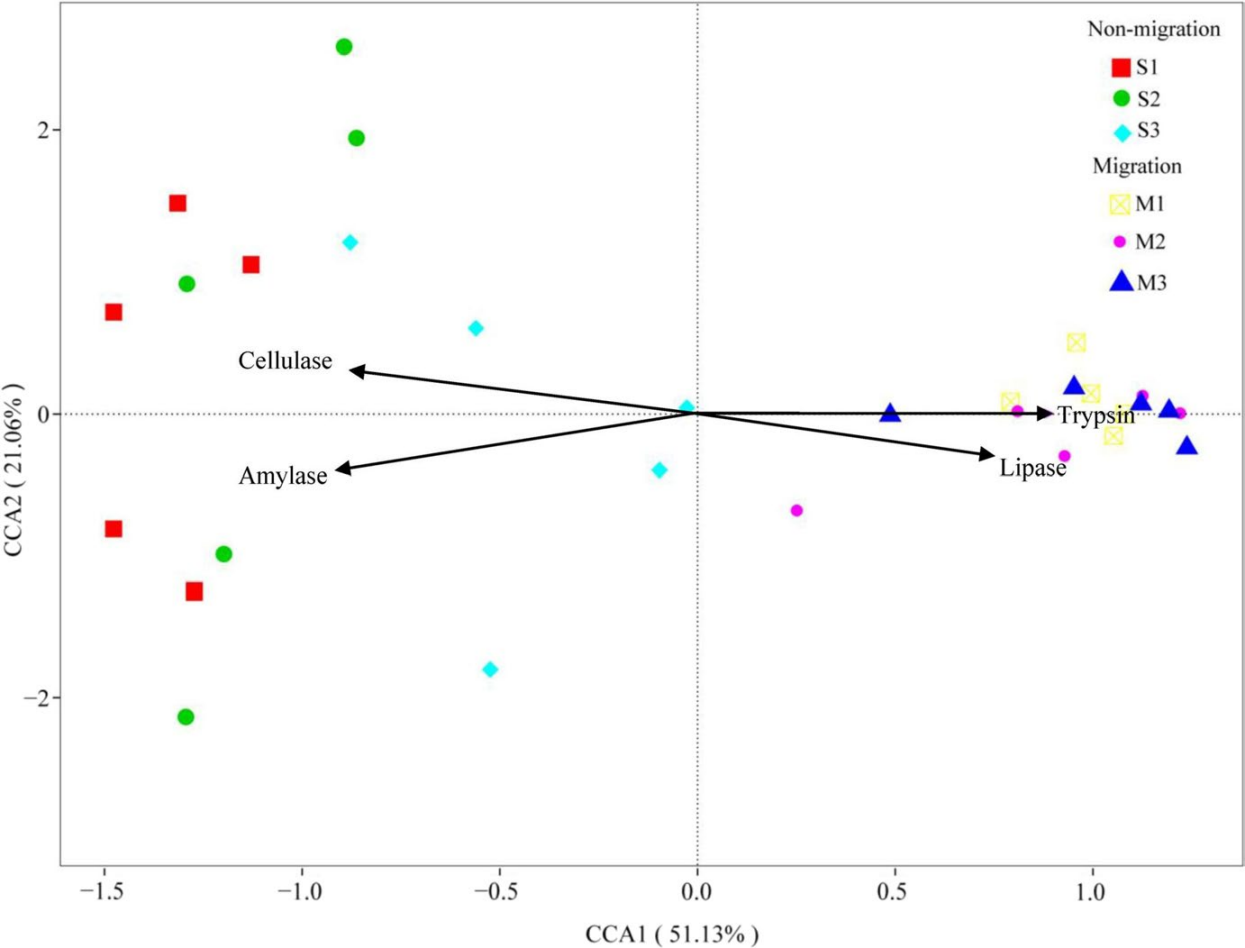
Canonical correspondence analysis (CCA) showed the correlation between the gut microbial compositions of the different group studied of *Megalobrama terminalis* and their enzyme activities

As shown in Table 3, the gut content digestive enzyme activity differed markedly in six groups. Cellulase activity was much higher (*p* < .05) in S1 than in M1, but no obvious difference with S1 and S2 group. And there was no significant difference among groups in migrated population. The trypsin activity in S1 group was not significantly different to S2 group but was lower than in S3 group. On the contrary, the amylase activity in S3 group was observed to be no significantly different to S2, but much higher than that in M1 group (*p* < .05). The amylase activity in M1 group was higher than that in S1 and S2 group (*p* < .05), but was not significantly different to M2 and M3 group. As a whole, cellulase and amylase activity in the gut content of nonmigration population was significantly higher than in migration population, while trypsin and lipase activity in migration population was much higher than in nonmigration population (Table 3). The gut microbial composition of each group had a close relationship with their metabolic enzymes (Figure 6). The gut microbiota composition of M1, M2, and M3 groups was more related to trypsin and lipase activity, and estranged from cellulase and amylase activities. In contrast, the gut microbial compositions of S1, S2, and S3 were correlated with cellulase and amylase activities.

**TABLE 3:** Gut content enzymes activities for the different group studied of *Megalobrama terminalis*

Distribution Zone		Gut content enzymes activities			
		Trypsin	Amylase	lipase	Cellulase
Nonmigration	S1	10.96 ± 2.27^a^	39.50 ± 4.78^b^	3.64 ± 1.12^a^	28.75 ± 5.04^a^
	S2	12.54 ± 3.14^a^	33.65 ± 3.11^b^	2.98 ± 0.97^a^	29.11 ± 6.49^a^
	S3	22.40 ± 2.32^b^	30.28 ± 4.66^b^	4.29 ± 0.75^ab^	20.12 ± 3.16^ab^
	Overall	16.43 ± 5.47	34.59 ± 6.25*	3.69 ± 1.09	24.15 ± 5.74*
Migration	M1	34.50 ± 4.04^c^	12.18 ± 1.21^a^	5.84 ± 1.87^b^	13.54 ± 2.19^a^
	M2	29.46 ± 3.11^c^	12.86 ± 3.06^a^	5.54 ± 1.47^b^	10.82 ± 1.92^a^
	M3	29.43 ± 3.65^c^	12.93 ± 3.69^a^	5.71 ± 1.49^b^	10.45 ± 1.57^a^
	Overall	31.13 ± 4.16*	12.66 ± 2.67	5.70 ± 1.51*	11.60 ± 2.27

Different superscript letters indicate significant differences in different groups, *p* < .05.

* Means significant difference between two populations, *p* < .05.

## 4 DISCUSSION

In recent years, it has been revealed that the fish gut microbial community plays a critical role in the host digestive system and immune system, attracting increasing attention (Liu et al., 2016). A previous study reported that the intestinal food item of black Amur bream in the developmental process was a transition phase in relation to the dietary shift, which exhibited the food preferences during gonad development period. (Xia et al., 2017). However, to the best of our knowledge, very few scientific literatures have reported the difference of the gut microbial population diversity between nonmigrated and migrated *M. terminalis* during spawning migration period. To investigate the relationship between gut microbiota and digestive functional shift of nonmigrated and migrated *M. terminalis*, we focused on changes in the gut microbiota and degradation enzymes activity of *M. terminalis* during its critical maturity stage. A detailed description of the gut microbiota and degradation enzymes activity of wild *M. terminalis* was provided in the present study. We observed and analyzed the gut microbiota community of *M. terminalis* sampled at different sites from estuary to main stem, which was regarded as migration routes of *M. terminalis* (Chen et al., 2020; Li et al., 2014; Xia et al., 2017). Significant difference of environmental factors was observed between estuary to main stem, especially in salinity and dissolved oxygen (Table 1). In recent studies of fish gut microbiota of many species, environmental factors have been shown to affect and shape the intestinal microbiota of fish (Sullam et al., 2012). Exogenous factors played important roles to affect the diversity of fish intestinal flora (Navarrete et al., 2012). The results indicated that *M. terminalis* population nonmigrated in estuary (fatten ground) had a higher alpha diversity than that of migrated population (Table 1), consistent with the previous research. Du et al. (2020) found significant differences in bacterioplankton community between the estuary and main stem. In combination with our result, there was a close interaction between the gut microbiome communities of *M. terminalis* population and the environmental microorganism.

Another interesting discovery is an obvious shift between the gut microbiota community of nonmigrated and migrated *M. terminalis* in Pearl River. PCoA revealed that fish gut bacterial communities at different sample sites formed two different clusters (Figure 2a), featured by the change of most abundant phylum of gut microbiota from Firmicutes to Proteobacteria (Figure 2b). Xia et al. (2017) reported a diet shift from Bacillariophyceae to Polyplacophora and Malacostraca during *M. terminalis* gonad development. Some researchers have shown that Proteobacteria are dominant in many carnivorous fishes (Kim et al., 2007; Liu et al., 2016; Merrifield et al., 2009). Ni et al. (2014) showed that Firmicutes was more advantageous than Proteobacteria in herbivorous grass carp. By fitting models of evolution, migratory species are evolving toward larger body size and higher fertility than nonmigratory species (Burns & Bloom, 2020). Studies indicated that not all individuals of migratory fish participated in the migration, and there were significant differences in sexual maturity, diet, and energy metabolism between migrated fish and nonmigrated fish (Secor, 1999). Meanwhile, migrated *M. terminalis* population in main stem had larger body size and higher gonadosomatic index than that of nonmigrated population (Table 1), which means that migrated population need more energy for spawning migration. As a result, it is necessary for migrated population to digest foods of higher nutritional value, which may be a key stimulus factor for the transition of gut microbiota community in *M. terminalis* to regulate the physiological capacity of digestion.

MetaStat results showed that abundance of *Massilia*, *Fibrobacter* in nonmigrated population was higher than that in migrated population, while abundance of *Kosakonia*, *Sphingomonas,* and *Stenotrophomonas* was much lower than that in migrated population (Figure 4). Cellulase and amylase activity in the gut content of nonmigration population was significantly higher than in migration population, while trypsin and lipase activity in migration population was much higher than in nonmigration population (Table 3). Our study revealed the four main enzyme activities which had a close relationship with the gut microbiome community (Figure 6). It has been reported that food digestion depends on the aid of gut symbiotic microorganisms to digest food and supply the energy to the host (Ray et al., 2012). Yokoe and Yasumasu (1964) mentioned that fish digested cellulose depends on the exogenous cellulose. Fibrobacteracea was shown to be a vital part of the gut flora of the herbivorous fish, *Ctenopharyngodon idelus* (Jiang et al., 2011). Gut cellulose activity is largely contributed by the gut microbiota. Meanwhile, research has revealed that Aeromonadaceae and Sphingomonadaceae occupied dominant positions in the freshwater carnivorous fish *Oncorhynchus mykiss* and *Siniperca Chuatsi* (Hovda et al., 2007; Kim et al., 2007; Merrifield et al., 2009). PICRUSt functional analysis of gut microbiota showed greater fructose and mannose metabolism and lower amino acid metabolism in migrated population compared with nonmigrated one (Figure 5). Lipid metabolism in migrated population was enhanced compared with that of nonmigrated population, seemingly explaining that the gut flora of *M. terminalis* was specialized for a diet shift and gonadal development during migration. This conclusion is in line with the results of research on gut microbiota and lipid metabolism in fish (Meng & Nie, 2019). An interesting phenomenon indicated that the gut microbiota plays a role in the endocrine system of the black Amur bream. This suggests that the gut microbiota may alter metabolism and the estrogen cycle during migration. Researches on mammals have shown that the significant variation of gut community was considered one of the factors affecting metabolism ability and sex hormone secretion changes (Goedert et al., 2018; Huang et al., 2020).

Above all, comparison of the microbiome composition of migrated and nonmigrated *M. terminalis* population indicated an obvious transformation, which is regarded as a critical factor response to gonadal development and dietary changes of *M. terminalis* during migration. Gut microbiota can be affected by multiple factors, for example, host diet, genetic, and environmental factors (Bolnick et al., 2014; Liu et al., 2016). The host developmental stage can influence individual gut floral diversity (Ley et al., 2008). Nevertheless, it is necessary to clarify how the bacterial coordination in the gut microbiota influences host behavior and physiology. Therefore, the ecology and physiology of the gut microbiota in fish should be an attractive field for future study.

## CONFLICT OF INTEREST

None declared.

## AUTHOR CONTRIBUTIONS

**Yaqiu Liu:** Conceptualization (lead); data curation (lead); resources (lead); visualization (equal); writing–original draft (lead). **Xinhui Li:** Funding acquisition (lead); supervision (equal). **Jie Li:** Funding acquisition (equal); investigation (supporting). **Weitao Chen:** Formal analysis (equal); writing–review and editing (lead).

## ETHICAL APPROVAL

The methods involving animals in this study were conducted in accordance with the Laboratory Animal Management Principles of China. All experimental protocols were approved by the Ethics Committee of the Pearl River Fisheries Research Institute, Chinese Academy of Fishery Sciences.

## Data Availability

All fastq files obtained from sequencing are publicly available from the NCBI Sequence Read Archive (SRA) database, (https: //www.ncbi.nlm.nih.gov/sra; Bioproject accession number PRJNA 656,398).
